# Inhibition of HSP90 attenuates porcine reproductive and respiratory syndrome virus production in vitro

**DOI:** 10.1186/1743-422X-11-17

**Published:** 2014-02-03

**Authors:** Jintao Gao, Shuqi Xiao, Xiaohong Liu, Liangliang Wang, Xiaoyu Zhang, Qianqian Ji, Yue Wang, Delin Mo, Yaosheng Chen

**Affiliations:** 1 State Key Laboratory of Biocontrol, School of Life Sciences, Sun Yat-sen University, Guangzhou 510006, PR China

**Keywords:** Porcine reproductive and respiratory syndrome virus, PRRSV, HSP90, Geldanamycin, 17-AAG, Antiviral

## Abstract

**Background:**

Porcine reproductive and respiratory syndrome virus (PRRSV) infection leads to substantial economic losses to the swine industry worldwide. However, no effective countermeasures exist to combat this virus infection so far. The most common antiviral strategy relies on directly inhibiting viral proteins. However, this strategy invariably leads to the emergence of drug resistance due to the error-prone nature of viral ploymerase. Targeting cellular proteins required for viral infection for developing new generation of antivirals is gaining concern. Recently, heat shock protein 90 (HSP90) was found to be an important host factor for the replication of multiple viruses and the inhibition of HSP90 showed significant antiviral effects. It is thought that the inhibition of HSP90 could be a promising broad-range antiviral approach. However, the effects of HSP90 inhibition on PRRSV infection have not been evaluated. In the current research, we tried to inhibit HSP90 and test whether the inhibition affect PRRSV infection.

**Methods:**

We inhibit the function of HSP90 with two inhibitors, geldanamycin (GA) and 17- allylamono-demethoxygeldanamycin (17-AAG), and down-regulated the expression of endogenous HSP90 with specific small-interfering RNAs (siRNAs). Cell viability was measured with alamarBlue. The protein level of viral N was determined by western blotting and indirect immunofluorescence (IFA). Besides, IFA was employed to examine the level of viral double-stranded RNA (dsRNA). The viral RNA copy number and the level of IFN-β mRNA were determined by quantitative real-time PCR (qRT-PCR).

**Results:**

Our results indicated that both HSP90 inhibitors showed strong anti-PRRSV activity. They could reduce viral production by preventing the viral RNA synthesis. These inhibitory effects were not due to the activation of innate interferon response. In addition, we observed that individual knockdown targeting HSP90α or HSP90β did not show dramatic inhibitory effect. Combined knockdown of these two isoforms was required to reduce viral infection.

**Conclusions:**

Our results shed light on the possibility of developing potential therapeutics targeting HSP90 against PRRSV infection.

## Background

Porcine reproductive and respiratory syndrome (PRRS) is characterized by severe reproductive failure in sows, and respiratory disease in young piglets, and causes huge economic losses in the swine industry [1]. The etiologic agent, porcine reproductive and respiratory syndrome virus (PRRSV) is an enveloped, single-stranded positive-sense RNA virus belonging to the Arteriviridae family [2] which includes equine arteritis virus (EAV), lactate dehydrogenase-elevating virus (LDV), and simian hemorrhagic fever virus (SHFV). Together with the Coronaviridae and Roniviridae families, Arteriviridae enters in the newly established order of the Nidovirales [3]. The genome of PRRSV is approximately 15 kb in length and encodes nine partially overlapping open reading frames (ORFs) designated ORF 1a, ORF 1b, and ORFs 2 to 7 [4]. As known, developments of vaccines and therapeutics are vital to the disease control. However, there are still no effective countermeasures available to treat this deadly viral disease. Development of effective antiviral strategies againt PRRSV infection is an urgent need [5,6].

Exposure of cells and tissues to extreme conditions such as heat, oxidative stress, heavy metals, UV irradiation and microbial/viral infection leads to selective transcription and translation of heat shock proteins (HSPs) [7,8]. HSPs are highly conserved and ubiquitous cytoprotective proteins, and involved in a multitude of cellular processes, including protein folding, refolding of stress-denatured protein, protein trafficking and degradation [9-11]. Based on their molecular weight, HSPs are divided into different classes: HSP100, HSP90, HSP70, HSP60, HSP40 and small HSPs [12]. HSP90 is one of highly abundant, essential, and conserved molecular chaperones present in eukaryotes [13]. Recently, HSP90 was shown to be an essential host factor for viral infection. It can be involved in different stages of the viral life cycle, including translocation [14,15], replication [12-14], gene expression [16], and virion morphogenesis [17]. Inhibition of HSP90 has been shown to reduce the replication of multiple viruses, such as vaccinia virus [18], hepatitis C virus [19], ebola virus [20], influenza virus [15], rotavirus [21], human cytomegalovirus [22], herpes simplex virus type 1 [23] and infectious bursal disease virus [24]. Accordingly, inhibition of HSP90 was regarded as a broad-range antiviral strategy [25]. However, the effects of HSP90 inhibition on PRRSV infection have not been evaluated. In current research, we inhibited HSP90 using specific functional inhibitors or RNA interference and evaluated the effects on PRRSV infection in vitro.

We found that the functional inhibition of HSP90 with two inhibitors, GA and 17-AAG, significantly reduced viral RNA synthesis, and attenuated final production. The addition of GA or 17-AAG did not induce the expression of IFN-β, indicating that these inhibitory effects are not due to the activation of innate interferon response.

Interestingly, no significant inhibitory effect was observed when individual knockdown of HSP90α or HSP90β. Combined knockdown of these two isoforms shown dramatic antiviral effect, suggesting that these two isoforms might have overlapping functions during PRRSV replication.

## Results

### The Cytotoxic Effects of HSP90 Inhibitors

**Figure 1:**
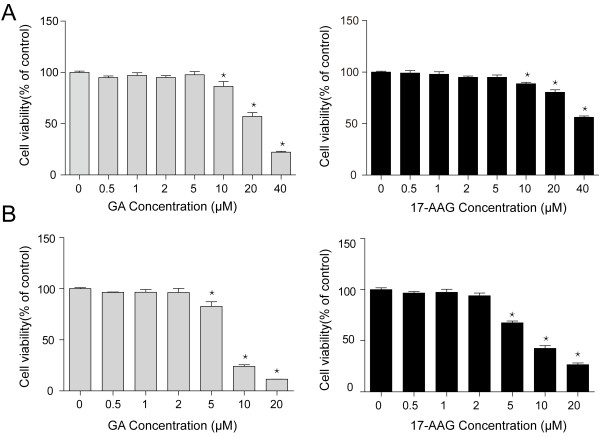
**The cell toxicity of GA and 17-AAG. (A)** MARC-145 cells and **(B)** PAMs were seeded in 96-well plates and then treated with serial concentration of drugs for 24 hours. Cell viability was measured using alamarBlue. Data are mean ± SD, n = 3, and significant differences compared with control are denoted by ^*^(P < 0.05).

The cytotoxic effects of two HSP90 inhibitors (GA and 17-AAG) on two types of PRRSV permissive cells, MARC-145 cells (Figure 1A) and primary porcine alveolar macrophages (PAMs) (Figure 1B), were examined by the alamarBlue cell viability assay (see Materials and methods). No significant toxicity was observed at concentrations of both inhibitors below 5 μM in MARC-145 cells (Figure 1A). PAMs were shown more sensitive to GA or 17-AAG and the minimal toxicity was found at concentrations below 2 μM (Figure 1B). Therefore, we performed future experiments with these two inhibitors at concentrations no higher than 5 μM in MARC-145 cells, and no higher than 2 μM in PAMs.

### HSP90 inhibitors attenuate the production of viral progeny

**Figure 2:**
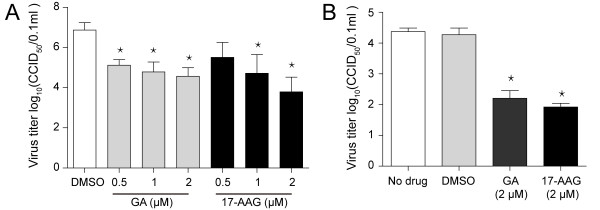
**HSP90 inhibitors reduced PRRSV production. (A)** MARC-145 cells and **(B)** PAMs were infected and treated with different concentrations of drugs. The culture supernatants were collected at 24 h.p.i and viral titers were determined by calculating CCID_50_. Data are mean ± SD, n = 3, and significant differences compared with DMSO (in MARC-145 cells) or no drug (in PAMs) treatment group, respectively, are denoted by ^*^(P < 0.05).

To tested the effects of two HSP90 inhibitors on the PRRSV production. PRRSV-infected MARC-145 cells or PAMs were treated with different concentrations of inhibitors. Viral titers were measured at 24 hous post infection (h.p.i). We observed that both HSP90 inhibitors reduced the production of PRRSV progeny in two cell types (Figure 2), and the inhibitory effects were found in a does-dependent manner (Figure 2A).

### HSP90 inhibitors decrease the viral protein level

**Figure 3:**
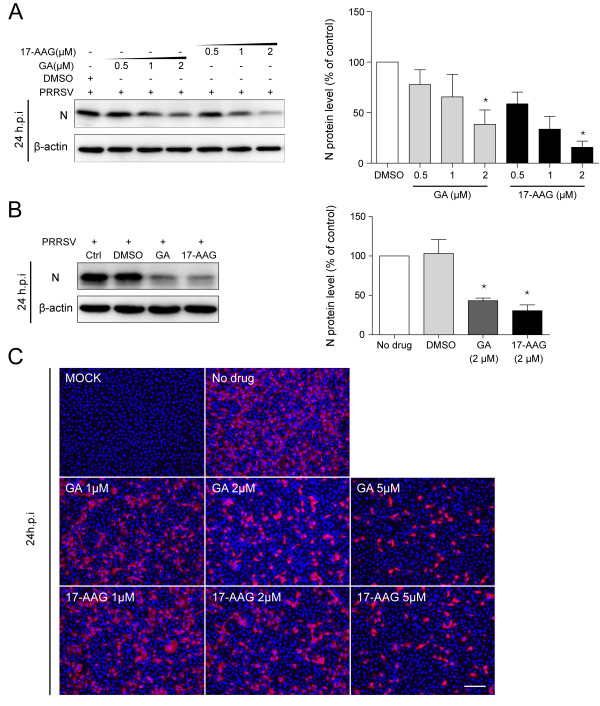
**GA and 17-AAG decreased the level of viral N protein.** PRRSV-infected cells were treated with chemicals at the concentration as indicated. Mock-infected cells were untreated. **(A)** MARC-145 cells and **(B)** PAMs were harvested at 24 h.p.i for western blotting analysis. The level of viral N protein was quantified by measuring band intensities and normalized with respect to the amount of β-actin. Data are mean ± SD, n = 3, and significant differences compared with DMSO (in MARC-145 cells) or no drug (in PAMs) treatment group, respectively, are denoted by ^*^(P < 0.05). **(C)** Inhibitors-treated MARC-145 cells were fixed at 24 h.p.i to detect viral N protein (red) by IFA. Nuclei were stained with Hoechst dye 33258 (blue). Bar, 200 μm.

We also evaluated the effects of the inhibitors on viral protein level. The expression of viral N protein in GA- or 17-AAG-treated cells was detected by western blotting and IFA. Similar inhibitory effects were found in viral protein level (Figure 3). GA or 17-AAG could decrease the level of viral N protein in a does-dependent manner (Figure 3A and 3C).

### GA or 17-AAG prevent the viral RNA synthesis

**Figure 4:**
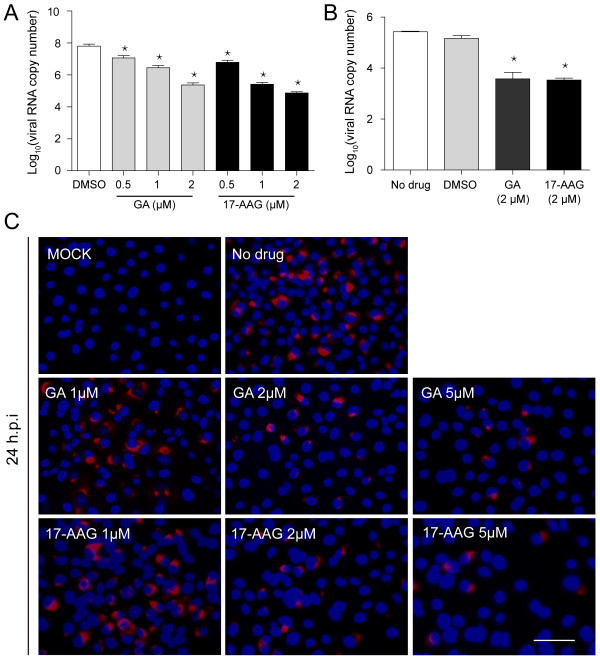
**HSP90 inhibitors prevented the viral RNA synthesis.** PRRSV-infected cells were treated with chemicals at the concentration as indicated. Mock-infected cells were untreated. **(A)** MARC-145 cells and **(B)** PAMs were harvested at 24 h.p.i. Total RNA was isolated and strand-specific qRT-PCR was performed to determine the viral full-length minus-stranded RNA copy number. Data are mean ± SD, n = 3, and significant differences compared with DMSO (in MARC-145 cells) or no drug (in PAMs) treatment group, respectively, are denoted by ^*^(P < 0.05). **(C)** Inhibitors-treated MARC-145 cells were fixed at 24 h.p.i to detect viral dsRNA (red) by IFA. Nuclei were stained with Hoechst dye 33258 (blue). Bar, 50 μm.

To investigate whether these inhibitory effects is due to the blockade of viral RNA synthesis, we performed strand-specific qRT-PCR [26] to measure the levels of PRRSV full-length minus-strand RNA. Our results showed that inhibitors treatment at the concentration of 2 μM could reduce the level of viral full-length minus-strand RNA in two cell types, dramatically (Figure 4A and 4B).

DsRNA can be generated during positive-sense RNA virus replication or transcription. The visualization of viral dsRNA has been shown to be a convenient approach to monitor the RNA synthesis of positive-sense RNA virus [27,28]. Therefore, IFA was performed to detect the level of viral dsRNA in inhibitors-treated MARC-145 cells. PRRSV dsRNA was shown mostly accumulating in the perinuclear region as observed in other positive-strand RNA viruses-infected cells. The amount of dsRNA was reduced in a dose-dependent manner in the presence of chemicals (Figure 4C).

### Interferon response is not triggered by HSP90 inhibitors

**Figure 5:**
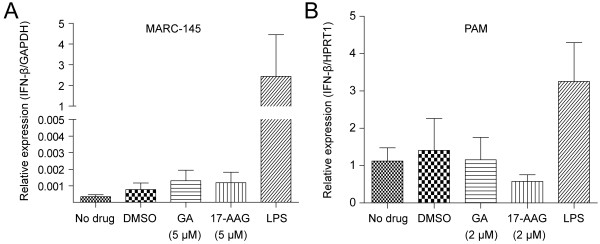
**GA or 17-AAG did not induce the IFN-β gene transcription. (A)** MARC-145 cells or **(B)** PAMs were treated with different concentrations of drugs for 10 hours and total cellular RNA was extracted for qRT-PCR analysis of IFN-β gene transcription.

The activation of type I interferon pathway is important for host against viral infection. To investigate whether GA- or 17-AAG-mediated inhibitory effects on PRRSV infection are due to the activation of interferon response, qRT-PCR was performed to measure the IFN-β mRNA level in inhibitors-treated cells. The IFN-β expression was not induced after inhibitors treatment in two cell types. As positive control, lipopolysaccharide (LPS) treatment could significantly elevate the level of IFN-β gene transcription (Figure 5).

### Combined knockdown of HSP90α and HSP90β is required to reduce the viral infection

**Figure 6:**
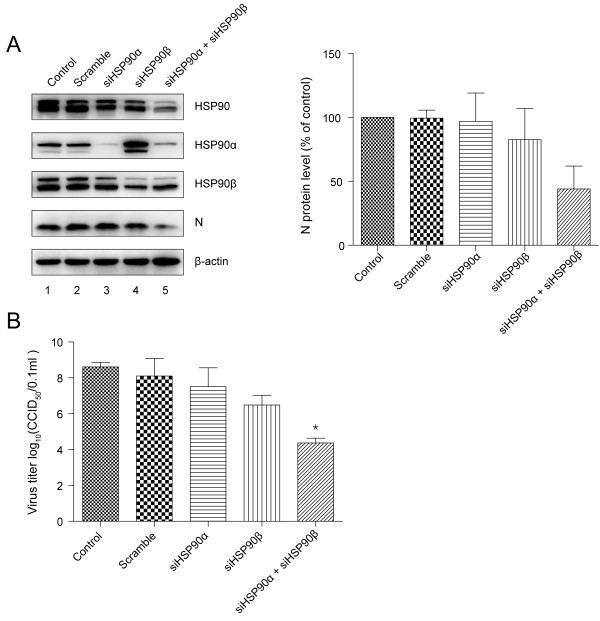
**The effects of knockdown of HSP90 on PRRSV infection. (A)** MARC-145 cells were transfected with different concentrations of siRNAs for 24 hours and subsequently infected with PRRSV. Cells were harvested at 24 h.p.i for western blotting analysis. The relative protein level of viral N was quantified by measuring band intensities and normalized with respect to the amount of β-actin. Meanwhile, **(B)** the culture supernatants were collected and viral titers were determined by calculating CCID_50_. Data are mean ± SD, n = 3, and significant differences compared with control are denoted by ^*^(P < 0.05).

The effects of knockdown of HSP90 on viral infection were also tested. In mammalian cells, there are two HSP90 isoforms, HSP90α and HSP90β, encoded by separate genes, and are essential for a multitude of cellular processes [29]. Therefore, we transfected siRNAs targeting each isoform or both into MARC-145 cells. Western blotting analysis showed that HSP90α and Hsp90β were down-regulated by siRNA, specifically (Figure 6A, lanes 3 and 4). Combined knockdown led to a dramatic reduction of viral N protein level and viral yields (Figure 6A, lanes 5; 6B). Interestingly, no significant antiviral effects were found when individual knockdown (Figure 6A, lanes 3 and 4; 6B). It may be due to the fact that individual knockdown could not lead to sufficient reduction of the total HSP90 (Figure 6A, lanes 3 and 4).

## Discussion

Infection with PRRSV causes a high mortality and leads to substantial economic losses to the swine industry worldwide. Vaccines have been developed to control PRRSV infection, but failed to provide sustainable disease control because of the viral immune evasion strategies and the antigenic heterogeneity of field strains [30]. Hence, the development effective antiviral strategies to combat PRRSV infection is an urgent necessity. The most common antiviral strategy relies on directly inhibiting viral proteins. However, this strategy invariably results in the emergence of drug resistance, as the virus can readily mutate to circumvent inhibition due to the error-prone nature of the ploymerase, even under conditions of combinatorial therapy targeting multiple viral proteins [31]. Targeting cellular proteins for developing new generation of antivirals is gaining concern [19-21,32]. HSP90 inhibitors, such as herbimycin A, radicicol, GA and its derivatives, characterized as effective anti-cancer therapeutics and several of which now in phase I and II clinical trials [33,34], have been shown to have strong antiviral activity. Strikingly, in the case of poliovirus, GA treatment did not led to the emergence of drug resistance within 10 passages [32]. Therefore, it is thought that HSP90 inhibitors could be promising broad-range antiviral agents. We have previously shown that HSP90 was elevated in PRRSV infected lungs relative to uninfected negative control (UNC) lungs based on transcriptome and proteome approaches [35,36], suggesting that HSP90 might be an important host factor for PRRSV infection as observed for other viruses. Therefore, we tried to inhibit HSP90 and test whether the inhibition could affect PRRSV infection.

In the present study, we found both inhibitors could block the synthesis of PRRSV RNA, and thus reduce viral infection in vitro. PAMs are known to be the primary host cellular target for PRRSV replication, thus the significant antiviral effects of these agents in these cells suggests that they might also be effective inhibitors against PRRSV in vivo. But it remains to be determined. Notably, GA or 17-AAG treatment could not induce IFN-β gene expression in both cell types. A previous research has showed that GA can inhibit the dsRNA- or virus-induced IFN-β gene expression in HeLa cells [37]. These results suggest that the anti-PRRSV activities performed by HSP90 inhibitors are not due to the activation of interferon response.

We also evaluated the effects of siRNA-mediated knockdown of HSP90 on PRRSV infection. The simultaneous depletion of both proteins led to a dramatic reduction of viral infection. However, no significant inhibitory effects were observed when individual knockdown, suggesting that these two isoforms might have overlapping functions during PRRSV infection. Interestingly, HSP90α was up-regulated after transfection with siRNA targeting HSP90β (Figure, 6A, lane 4), which is consistent with a previous research [38], indicating a compensatory up-regulation. But the corresponding augmentation of HSP90β after transfection of siHSP90α was not observed (Figure, 6A, lane 3), which may be due to the fact that HSP90β is generally constitutive and not sensitive to a great variety of stimuli [29,39].

As known, HSP90 can be involved in different stages of the viral life cycle. Our results showed that the PRRSV RNA synthesis was prevented by GA and 17-AAG treatment, suggesting that HSP90 is somehow involved in supporting the PRRSV replication. In addition, lower levels of viral protein and viral production were found. Hence, it is not excluded that HSP90 may also be involved in the PRRSV life cycle at the steps of protein synthesis and budding. Notably, the inhibitory effects observed in this study are not due to inhibition in virus adsorption or entry since in all exprements, the inhibitors were added at 1 h.p.i, when PRRSV has been internalized in host cells [40]. The addition of inhibitors, even at 4 h.p.i, also showed significant inhibitory effects (data not shown). However, these results could not exclude the possibility that HSP90 could regulate PRRSV infection in absorption and internalization.

The exact roles that HSP90 plays during PRRSV infection remain to be determined. HSP90 can regulate viral infection by modulating the host processes or interacting with viral proteins directly [21,25]. Therefore, further study will be mainly performed in our laboratory in two aspects: (I) identification of PRRSV protein associated with HSP90 directly; (II) investigation whether HSP90 is exploited by PRRSV to regulate cellular processes for its benefit.

## Conclusions

Our results provide some insight into possible future development of potential therapeutics against PRRSV infection.

## Methods

### Cell culture

MARC-145 cultured in Dulbecco’s modified Eagle’s medium (DMEM) containing 10% Fetal Bovine Serum (FBS) were maintained at 37°C with 5% CO_2_.

PAMs were obtained postmortem lung lavage of 8-week-old specific pathogen free (SPF) pigs, and maintained in RPMI 1640 medium containing 10% FBS and penicillin/streptomycin.

### Chemicals and antibodies

GA and 17-AAG obtained from Invivogen (San Diego, CA, USA) were re-suspended in DMSO.

Rabbit anti-actin, anti-HSP90α, anti-HSP90β, anti-HSP90 antibodies were obtained from Cell Signaling Technology (Beverly, MA, USA). Mouse anti-PRRSV N protein antibody was obtained from Jeno Biotech Inc (Chuncheon, South Korea). Mouse monoclonal antibody specific for dsRNA (J2) was purchased from Scicons (Hungary).

### Virus infection and chemicals treatment

Cells were infected with PRRSV strain CH-1a (the first type 2 PRRSV strain isolated in China, kindly provided by Dr. Guihong Zhang in South China Agricultural University, China) at an MOI of 0.1. At 1 h.p.i, cells were untreated (no drug) or treated with GA, 17-AAG, or DMSO. The 50% cell culture infection dose (CCID_50_) was calculated at 24 h.p.i by the Reed-Muench method.

Cells were treated with different concentrations of drugs or LPS (at the final concentration of 10 μg/ml, as positive control) for 10 hours and harvested for IFN-β transcription analysis.

### Cell viability assay

Cells were seeded into 96-well plates. After 24 hours treatment with GA or 17-AAG, cells were incubated at 37°C with fresh DMEM medium containing 10% alamarBlue (Invitrogen, Carlsbad, CA, USA) for 4 hours in accordance with the manufacturer’s protocol. Fluorescence was monitored at 570 nm excitation and 590 nm emission wavelengths using a Synergy 2 Multi-Mode Microplate reader (BioTek). The fluorescence was directly proportional to the number of living cells in culture.

### SiRNAs and transfection

SiRNAs were obtained from Ribobio (Guangzhou, China), and used at a 50 nM final concentration. MARC-145 cells grown to approximately 30-50% confluence in 6-well plates were transfected with no siRNA (control), scramble siRNA, siHSP90α (50 nM), siHSP90β (50 nM), or both siHSP90α (25 nM) and siHSP90β (25 nM) using lipofectamine 2000 (Invitrogen) according to the manufacturer’s instruction.

### Quantitative RT-PCR assays

Cells were harvested and total RNA was isolated at 24 h.p.i with TRIzol™ reagent (Invitrogen) according to the manufacturer’s instruction. The synthesis of the first strand of cDNA was performed using a reverse transcription kit (Promega, Madison, WI, USA). Quantitative PCR was performed with SYBR Premier Dimer Eraser™ kit (TaKaRa, China) using LightCycler® 480 Real-Time PCR System and analyzed with LightCycler® 480 software (Roche). The detection of full-length minus-strand RNA was performed as described in previous study [26]. Specific primers used for amplification of IFN-β in MARC-145 cells (mIFN-β) [41] or in PAMs (pIFN-β) [42] was the following: mIFN-β-F, 5′-TAAGCAGCTGCAGCAGTTCCAGAAG-3′; mIFN-β-R,5′-GTCTCATTCCAGCCAGTGCT-3′; pIFNB1-F:5′-TGCAACCACCACAATTCC-3′; pIFNB1-R:5′-CTGAGAATGCCGAAGATCTG-3′. The mRNA of GAPDH or HPRT1 served as internal reference.

### Western blotting

Cell pellets were lysed in cell lysis buffer (Beyotime Biotechnol, Shanghai, China) containing 1 mM phenylmethyl-sulfonylfluoride (PMSF) and samples were boiled for 5 minutes. About 25 μg of protein was separated by sodium dodecyl sulfate- polyacrylamide gel electrophoresis (SDS-PAGE) and blotted onto a polyvinyl difluoride (PVDF) membrane. After blotting, the membrane was blocked with 5% nonfat dry milk in Tris-Buffered Saline containing Tween 20 (TBST) for 2 hours and incubated overnight at 4°C with primary antibody. The membrane were then washed in TBST and incubated for 1 hour with the HRP-conjugated secondary antibodies (CST). Imaging of the blot was performed with super signal west pico chemiluminescence substrate (Pierce, IL, USA) using Image Station 4000 mm PRO System (Kodak). Protein band intensities were measured by Image Station 4000 mm PRO software. The control group was set as 100 to allow comparisons.

### Indirect immunofluorescence assay

MARC-145 cells grown on glass slides were fixed with 4% paraformaldehyde in phosphate-buffered saline (PBS) at room temperature for 10 minutes. After being washed three times with PBS, the cells were permeabilized for 15 minutes at room temperature with PBS containing 0.5% Triton X-100 and blocked with PBS containing 1% Bovine serum albumin (BSA) for 30 minutes at room temperature. The cells were incubated with primary antibody in PBS containing 1% BSA at 4°C overnight. The cells were then washed three times with PBS and incubated with AF555-conjugated anti-mouse IgG (CST) in PBS containing 1% BSA at room temperature for 60 minutes. Nuclei were stained with Hoechst dye 33258 (Sigma-Aldrich, MO, USA) for 4 minutes at room temperature. Finally, the cells were washed three times with PBS and observed with ELYRA P.1 prototype system (Carl Zeiss).

### Statistical analysis

Data were presented as means ± standard errors and resulted from three independent experiments. Statistical significance was determined by Student’s t test. A P value < 0.05 was considered statistically significant.

## Competing interests

The authors declare that they have no competing interests.

## Authors’ contributions

JG and SX conceived and designed the study. JG and SX performed the experiments, analyzed the data, and wrote the manuscript. XL, LW, XZ, QJ, YW, DM coordinated the study. YC contributed to the interpretation of the results and took part in the critical revision of the manuscript. All authors read and approved the final manuscript.

## References

[B1] Neumann EJ Kliebenstein JB Johnson CD Mabry JW Bush EJ Seitzinger AH Green AL Zimmerman JJ Assessment of the economic impact of porcine reproductive and respiratory syndrome on swine production in the United States J Am Vet Med Assoc 2005 227 3 385 392 10.2460/javma.2005.227.385 16121604

[B2] Meulenberg JJ Hulst MM de Meijer EJ Moonen PL den Besten A de Kluyver EP Wensvoort G Moormann RJ Lelystad virus, the causative agent of porcine epidemic abortion and respiratory syndrome (PEARS), is related to LDV and EAV Virol 1993 192 1 62 72 10.1006/viro.1993.1008 PMC7173055 8517032

[B3] Cavanagh D Nidovirales: a new order comprising Coronaviridae and Arteriviridae Arch Virol 1997 142 3 629 633 9349308

[B4] Dea S Gagnon C Mardassi H Pirzadeh B Rogan D Current knowledge on the structural proteins of porcine reproductive and respiratory syndrome (PRRS) virus: comparison of the North American and European isolates Arch Virol 2000 145 4 659 688 10.1007/s007050050662 10893147 PMC7087215

[B5] Cho JG Dee SA Porcine reproductive and respiratory syndrome virus Theriogenology 2006 66 3 655 662 10.1016/j.theriogenology.2006.04.024 16730057

[B6] Zhou L Yang H Porcine reproductive and respiratory syndrome in China Virus Res 2010 154 1 31 37 20659506 10.1016/j.virusres.2010.07.016

[B7] Morimoto RI Proteotoxic stress and inducible chaperone networks in neurodegenerative disease and aging Genes Dev 2008 22 11 1427 1438 10.1101/gad.1657108 18519635 PMC2732416

[B8] Pirkkala L Nykänen P Sistonen L Roles of the heat shock transcription factors in regulation of the heat shock response and beyond FASEB J 2001 15 7 1118 1131 10.1096/fj00-0294rev 11344080

[B9] Hartl FU Bracher A Hayer-Hartl M Molecular chaperones in protein folding and proteostasis Nature 2011 475 7356 324 332 10.1038/nature10317 21776078

[B10] Bukau B Weissman J Horwich A Molecular chaperones and protein quality control Cell 2006 125 3 443 451 10.1016/j.cell.2006.04.014 16678092

[B11] Young JC Agashe VR Siegers K Hartl FU Pathways of chaperone-mediated protein folding in the cytosol Nat Rev Mol Cell Biol 2004 5 10 781 791 10.1038/nrm1492 15459659

[B12] Hartl FU Molecular chaperones in cellular protein folding Nature 1996 381 6583 571 580 10.1038/381571a0 8637592

[B13] Picard D Heat-shock protein 90, a chaperone for folding and regulation Cell Mol Life Sci 2002 59 10 1640 1648 10.1007/PL00012491 12475174 PMC11337538

[B14] Momose F Naito T Yano K Sugimoto S Morikawa Y Nagata K Identification of Hsp90 as a stimulatory host factor involved in influenza virus RNA synthesis J Biol Chem 2002 277 47 45306 45314 10.1074/jbc.M206822200 12226087

[B15] Chase G Deng T Fodor E Leung BW Mayer D Schwemmle M Brownlee G Hsp90 inhibitors reduce influenza virus replication in cell culture Virology 2008 377 2 431 439 10.1016/j.virol.2008.04.040 18570972

[B16] Basha W Kitagawa R Uhara M Imazu H Uechi K Tanaka J Geldanamycin, a potent and specific inhibitor of Hsp90, inhibits gene expression and replication of human cytomegalovirus Antivir Chem Chemother 2005 16 2 135 146 15889536 10.1177/095632020501600206

[B17] Jindal S Young RA Vaccinia virus infection induces a stress response that leads to association of Hsp70 with viral proteins J Virol 1992 66 9 5357 5362 1501279 10.1128/jvi.66.9.5357-5362.1992 PMC289091

[B18] Hung J-J Chung C-S Chang W Molecular chaperone Hsp90 is important for vaccinia virus growth in cells J Virol 2002 76 3 1379 1390 10.1128/JVI.76.3.1379-1390.2002 11773412 PMC135870

[B19] Nakagawa S-i Umehara T Matsuda C Kuge S Sudoh M Kohara M Hsp90 inhibitors suppress HCV replication in replicon cells and humanized liver mice Biochem Biophys Res Commun 2007 353 4 882 888 10.1016/j.bbrc.2006.12.117 17196931

[B20] Smith DR McCarthy S Chrovian A Olinger G Stossel A Geisbert TW Hensley LE Connor JH Inhibition of heat-shock protein 90 reduces Ebola virus replication Antiviral Res 2010 87 2 187 194 10.1016/j.antiviral.2010.04.015 20452380 PMC2907434

[B21] Dutta D Bagchi P Chatterjee A Nayak MK Mukherjee A Chattopadhyay S Nagashima S Kobayashi N Komoto S Taniguchi K The molecular chaperone heat shock protein-90 positively regulates rotavirus infection Virology 2009 391 2 325 333 10.1016/j.virol.2009.06.044 19628238

[B22] Evers DL Chao C-F Zhang Z Huang E-S 17-allylamino-17-(demethoxy) geldanamycin (17-AAG) is a potent and effective inhibitor of human cytomegalovirus replication in primary fibroblast cells Arch Virol 2012 157 10 1971 1974 10.1007/s00705-012-1379-7 22711259

[B23] Li Y-H Tao P-Z Liu Y-Z Jiang J-D Geldanamycin, a ligand of heat shock protein 90, inhibits the replication of herpes simplex virus type 1 in vitro Antimicrob Agents Chemother 2004 48 3 867 872 10.1128/AAC.48.3.867-872.2004 14982777 PMC353133

[B24] Yuan W Zhang X Xia X Sun H Inhibition of infectious bursal disease virus infection by artificial microRNAs targeting chicken heat-shock protein 90 J Gen Virol 2012 93 Pt 4 876 879 22238234 10.1099/vir.0.039172-0

[B25] Geller R Taguwa S Frydman J Broad action of Hsp90 as a host chaperone required for viral replication Biochim Biophys Acta (BBA)-Mol Cell Res 2012 1823 3 698 706 10.1016/j.bbamcr.2011.11.007 PMC3339566 22154817

[B26] Zhang Y-J Stein DA Fan S-M Wang K-Y Kroeker AD Meng X-J Iversen PL Matson DO Suppression of porcine reproductive and respiratory syndrome virus replication by morpholino antisense oligomers Vet Microbiol 2006 117 2 117 129 16839712 10.1016/j.vetmic.2006.06.006 PMC7117520

[B27] Westaway EG Khromykh AA Mackenzie JM Nascent Flavivirus RNA Colocalized *in Situ* with Double-Stranded RNA in Stable Replication Complexes Virology 1999 258 1 108 117 10.1006/viro.1999.9683 10329573

[B28] Targett-Adams P Boulant S McLauchlan J Visualization of double-stranded RNA in cells supporting hepatitis C virus RNA replication J Virol 2008 82 5 2182 2195 10.1128/JVI.01565-07 18094154 PMC2258944

[B29] Csermely P Schnaider T Prohászka Z Nardai G The 90-kDa molecular chaperone family: structure, function, and clinical applications. A comprehensive review Pharmacol Ther 1998 79 2 129 168 10.1016/S0163-7258(98)00013-8 9749880

[B30] Kimman TG Cornelissen LA Moormann RJ Rebel JM Stockhofe-Zurwieden N Challenges for porcine reproductive and respiratory syndrome virus (PRRSV) vaccinology Vaccine 2009 27 28 3704 3718 10.1016/j.vaccine.2009.04.022 19464553

[B31] Pillay D Zambon M Antiviral drug resistance BMJ: Br Med J 1998 317 7159 660 10.1136/bmj.317.7159.660 PMC1113839 9728000

[B32] Geller R Vignuzzi M Andino R Frydman J Evolutionary constraints on chaperone-mediated folding provide an antiviral approach refractory to development of drug resistance Genes Dev 2007 21 2 195 205 10.1101/gad.1505307 17234885 PMC1770902

[B33] Goetz MP Toft D Reid J Ames M Stensgard B Safgren S Adjei AA Sloan J Atherton P Vasile V Phase I trial of 17-allylamino-17-demethoxygeldanamycin in patients with advanced cancer J Clin Oncol 2005 23 6 1078 1087 10.1200/JCO.2005.09.119 15718306

[B34] Whitesell L Lindquist SL HSP90 and the chaperoning of cancer Nat Rev Cancer 2005 5 10 761 772 10.1038/nrc1716 16175177

[B35] Xiao S Jia J Mo D Wang Q Qin L He Z Zhao X Huang Y Li A Yu J Understanding PRRSV infection in porcine lung based on genome-wide transcriptome response identified by deep sequencing PLoS One 2010 5 6 e11377 10.1371/journal.pone.0011377 20614006 PMC2894071

[B36] Xiao S Wang Q Jia J Cong P Mo D Yu X Qin L Li A Niu Y Zhu K Research Proteome changes of lungs artificially infected with H-PRRSV and N-PRRSV by two-dimensional fluorescence difference gel electrophoresis Virol J 2010 7 1 1 17 10.1186/1743-422X-7-1 20504321 PMC2887434

[B37] Iwamura T Yoneyama M Yamaguchi K Suhara W Mori W Shiota K Okabe Y Namiki H Fujita T Induction of IRF-3/-7 kinase and NF-κB in response to double-stranded RNA and virus infection: common and unique pathways Genes Cells 2001 6 4 375 388 10.1046/j.1365-2443.2001.00426.x 11318879

[B38] Chatterjee M Jain S Stühmer T Andrulis M Ungethüm U Kuban R-J Lorentz H Bommert K Topp M Krämer D STAT3 and MAPK signaling maintain overexpression of heat shock proteins 90α and β in multiple myeloma cells, which critically contribute to tumor-cell survival Blood 2007 109 2 720 728 10.1182/blood-2006-05-024372 17003370

[B39] Chang Y-S Lo C-W Sun F-C Chang MD-T Lai Y-K Differential expression of Hsp90 isoforms in geldanamycin-treated 9 L cells Biochem Biophys Res Commun 2006 344 1 37 44 10.1016/j.bbrc.2006.03.157 16630568

[B40] Delrue I Van Gorp H Van Doorsselaere J Delputte PL Nauwynck HJ Susceptible cell lines for the production of porcine reproductive and respiratory syndrome virus by stable transfection of sialoadhesin and CD163 BMC Biotechnol 2010 10 1 48 10.1186/1472-6750-10-48 20587060 PMC2908558

[B41] Hu H Zhang X Zhang H Wen G Zhang Q Li X Fang W Porcine reproductive and respiratory syndrome virus inhibition of interferon-β transcription by IRF3-independent mechanisms in MARC-145 cells in early infection Vet Immunol Immunopathol 2013 156 1 135 140 24148827 10.1016/j.vetimm.2013.09.015 PMC7112902

[B42] Loving CL Brockmeier SL Sacco RE Differential type I interferon activation and susceptibility of dendritic cell populations to porcine arterivirus Immunol 2007 120 2 217 229 10.1111/j.1365-2567.2006.02493.x PMC2265861 17116172

